# Quantitative Angiographic Assessment of Aortic Regurgitation After Transcatheter Implantation of the Venus A-valve: Comparison with Other Self-Expanding Valves and Impact of a Learning Curve in a Single Chinese Center

**DOI:** 10.5334/gh.1046

**Published:** 2021-08-04

**Authors:** Rutao Wang, Hideyuki Kawashima, Darren Mylotte, Liesbeth Rosseel, Chao Gao, Jean-Paul Aben, Mahmoud Abdelshafy, Yoshinobu Onuma, Jian Yang, Osama Soliman, Ling Tao, Patrick W. Serruys

**Affiliations:** 1 Department of Cardiology, Xijing Hospital, Xi’an, CN; 2 Department of Cardiology, National University of Ireland, Galway (NUIG), Galway and CORRIB Research Center for Advanced Imaging and Core laboratory, IE; 3 Department of Cardiology, Radboud University Medical Center, Nijmegen, NL; 4 Amsterdam UMC, University of Amsterdam, Heart Center, Department of Clinical and Experimental Cardiology, Amsterdam Cardiovascular Sciences, Amsterdam, NL; 5 Pie Medical Imaging, Maastricht, NL; 6 Department of Cardiovascular Surgery, Xijing Hospital, Xi’an, CN; 7 NHLI, Imperial College London, London, UK

**Keywords:** aortic stenosis, self-expanding valve, aortic regurgitation, transcatheter aortic valve replacement, transcatheter heart valve

## Abstract

**Objectives:**

We aimed to compare the quantitative angiographic aortic regurgitation (AR) into the left ventricular out flow tract (LVOT-AR) of five different types of transcatheter self-expanding valves and to investigate the impact of the learning curve on post-TAVR AR.

**Background:**

Quantitative video densitometric aortography is an objective, accurate, and reproducible tool for assessment of AR following TAVR.

**Methods and results:**

This retrospective academic core-lab analysis, analyzed 1150 consecutive cine aortograms performed immediately post-TAVR. Quantitative angiographic AR of post-procedural aortography in 181 consecutive patients, who underwent TAVR with the Venus A-valve in a single Chinese center, were compared to the results of Evolut Pro, Evolut R, CoreValve, (Medtronic, Dublin, Ireland) and Acurate Neo (Boston Scientific, Massachusetts, US) transcatheter heart valves (THVs), from a previously published pooled database. Among the 181 aortograms of patients treated with the Venus A-Valve, 113 (62.4%) were analyzable for quantitative assessment of AR. The mean LVOT-AR was 8.9% ± 10.0% with 14.2% of patients having moderate or severe AR in the Venus A-valve group. No significant difference in mean LVOT-AR was observed between Evolut Pro, Evolut R, Acurate Neo, and Venus A-valve. The incidence of LVOT-AR >17%, which correlates with echocardiographic derived ≥ moderate AR, with the Evolut Pro was lower than with the Venus A-valve (5.3% vs. 14.2%, p = 0.034), but was not different from the Evolut R (5.3% vs. 8.8%, p = 0.612), or the Acurate Neo (5.3% vs. 11.3% p = 0.16) systems. A landmark analysis after recruitment of the first half of patients treated with the Venus A valve (N = 56), showed a significantly lower mean LVOT-AR in the second half of the series (11.3% ± 11.9% vs. 6.5% ± 7.1%, p = 0.011). The incidence of LVOT-AR >17% in the latest 57 cases was also numerically lower (7.0% vs. 21.4%, p = 0.857) and compared favorably with the best in class of the self-expanding valves.

**Conclusion:**

The Venus A-valve has comparable mean LVOT-AR to other self-expanding valves but has a higher rate of moderate or severe AR than the Evolut Pro THV. However, after completion of a learning phase, results improved and compared favorably with the best in class of the commercially available self-expanding valves. These findings should be confirmed in prospective randomized comparisons of AR between different THVs.

## Introduction

Quantitative video densitometric assessment of aortic regurgitation (AR) after transcatheter aortic valve replacement (TAVR) has been validated in silico, in vivo, in clinical trials and real-world populations [1]. Moderate or severe AR has been associated with increased long-term mortality and constitutes an important mechanistic endpoint in trials comparing different transcatheter heart valve (THV) designs [2], in which the risk of AR with self-expanding valves (SEV) and balloon expandable valves (BEV) are comparatively evaluated [3,4].

AR following TAVR is mostly paravalvular leak (PVL) in nature. AR severity depends on the interaction between anatomical characteristics of the native aortic valve (bicuspid leaflet, elliptical annulus, calcified cups…, etc.), on the type of THV platform and on the implantation technique [5,6,7]. Modifications in the design of THVs, such as radial force, sealing skirt, frame composition or size of struts, have the potential to influence a THV’s anti-PVL sealing capacity. A wide variation in AR severity among different THVs has been described (2%-30% AR in the left ventricular outflow tract according to the aortographic criteria [LVOT-AR] >17% for moderate AR), with lower degree of LVOT-AR observed in THVs that feature an anti-PVL skirt [8,9]. In this study, the first data, stemming from a single Chinese center (Xijing hospital, Xi’an, China), quantitative video densitometric assessment of AR in TAVR patients with the Venus A-valve (Venus Medtech Inc., Hangzhou, China) are presented, and compared to four other commercially available self-expanding THVs.

## Methods

This is a retrospective, single-center analysis of consecutive aortograms in 181 consecutive TAVR patients treated with the Venus A-valve at the Xijing hospital. Video densitometry analysis of AR was performed by two physicians of the Xijing hospital (RW and CG) and remotely supervised by an independent core laboratory (HK, MA, YO, PWS, OS of the CORRIB Corelab, NUIG, in Galway) using the CAAS A-valve 2.0.2 software (Pie Medical Imaging BV, Maastricht, the Netherlands). This quantitative assessment of the AR from the aorta into the left ventricle outflow tract is reported as the LVOT-AR parameter; the results are expressed in percentages and quantify the fraction of AR defined as the ratio between the area under the time-density curves assessed by video densitometry in the LVOT (region of interest, ROI) and in the aortic root (reference area) during a conventional aortography. Technical details of video densitometry analysis and validation in silico, in vivo, in animal models as well as in clinical correlations with magnetic resonance imaging, transthoracic and transesophageal echocardiography have been extensively reported in literature [10,11,12,13,14]. Post-implantation balloon dilatation improvement in AR has also been quantitatively documented in a series of 61 patients [15]. Importantly, video densitometry-derived AR has proven to be a predictor of long-term prognosis after TAVR, with a >17 % threshold of AR identifying those at risk of long-term mortality [10,15]. The results of quantitative analyses of Evolut Pro, Evolut R, CoreValve (Medtronic, Dublin, Ireland), and Acurate Neo (Boston Scientific, Massachusetts, USA) were retrieved from a published pooled database [8].

The Venus A-valve is a self-expanding THV, consisting of a nitinol stent frame with supra-annular porcine pericardial leaflets, but without an anti-leak skirt, and was granted the China Food and Drug Administration approval in 2017. We aimed to assess the video densitometric AR with the Venus A-valve and to compare to the performance of four commercially available different types of SEVs. All procedures and evaluation were performed according to Good Clinical Practice and in accordance with the Declaration of Helsinki.

## Statistical Analysis

**Figure 1:**
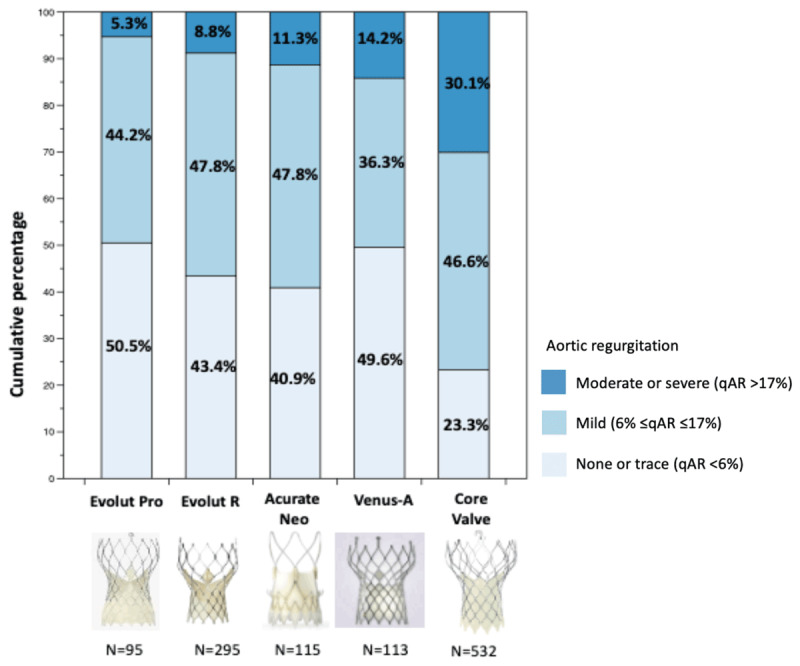
Cumulative percentage of different degrees of post-TAVR AR by video densitometric assessment. AR: aortic valve regurgitation; TAVR: transcatheter aortic valve replacement.

Continuous variables were reported as mean ± standard deviations. Comparison of LVOT-AR was performed using one-way analysis of variance and two-by-two comparisons using the post-hoc Bonferroni test. A landmark analysis, comparing LVOT-AR between the first 56 cases and latest 57 cases with the Venus A-valve was performed using an unpaired student t–test. The continuous variable ‘LVOT-AR’ was stratified into categorical variables according to the following pre-determined threshold criteria: 1) none or trace (<6%); 2) mild (6% to ≤17%); and 3) moderate or severe (>17%) (Figure 1) [8]. The proportion of patients with moderate or severe AR (LVOT-AR >17%) was compared using the chi-square test. A p value of <0.05 was considered indicative of statistical significance. Statistical analyses were performed with SPSS version 25.0 (IBM, Armonk, New York, USA).

## Results

**Figure 2:**
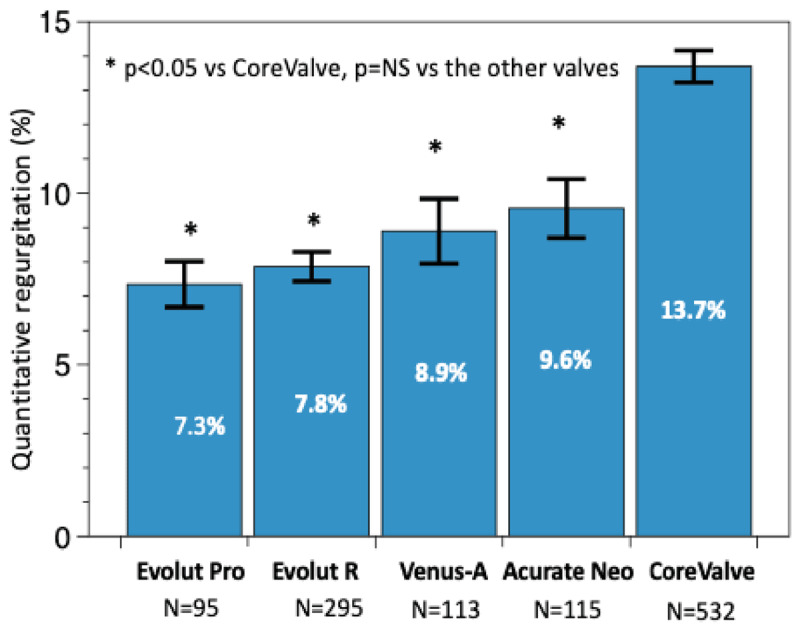
Comparison of LVOT-AR after TAVR among the five THVs. Bars denote the mean regurgitation values, and error bars denote standard errors of the mean. LVOT-AR: quantitative aortic regurgitation in the left ventricular outflow tract; TAVR: transcatheter aortic valve replacement; THV: transcatheter heart valve.

**Figure 3:**
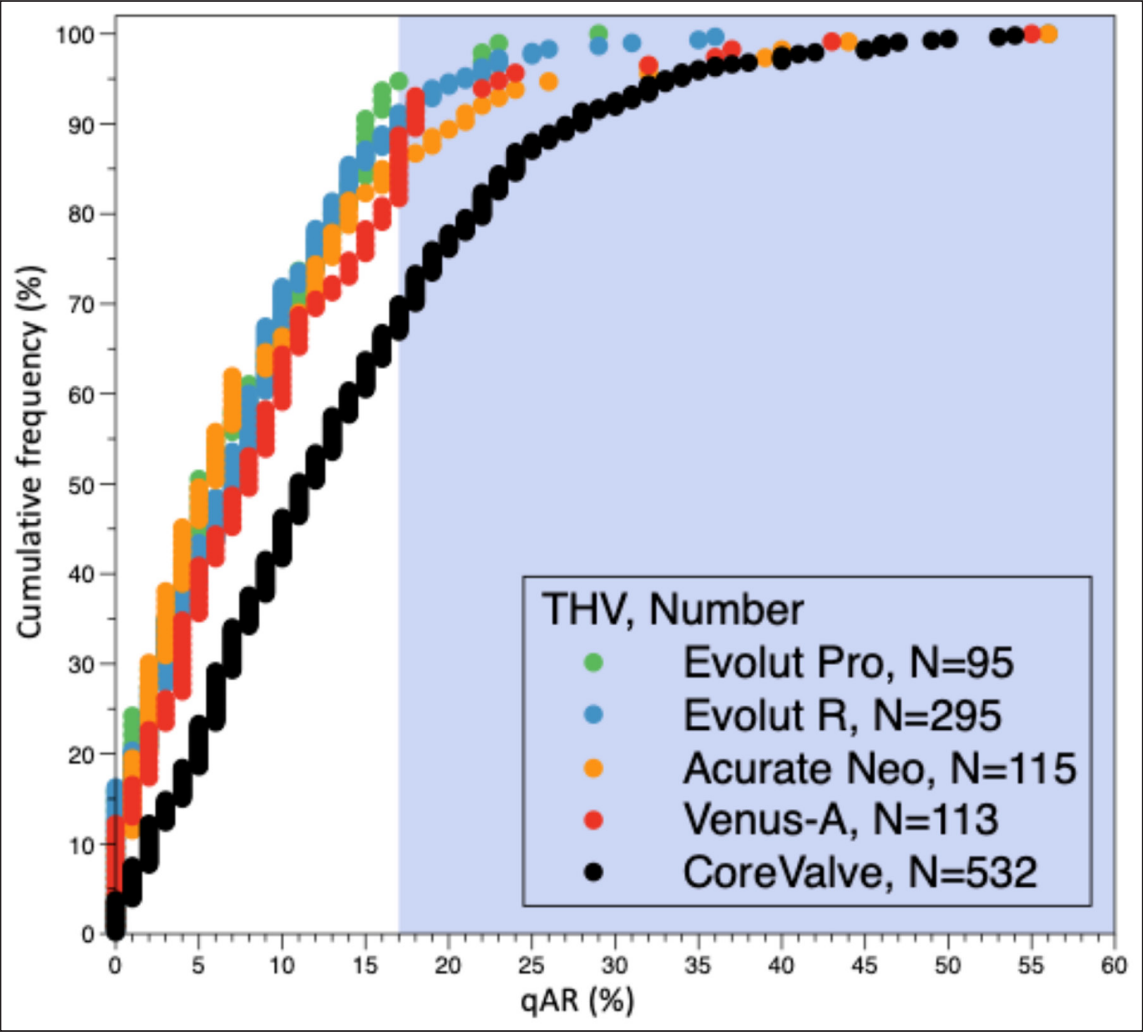
Cumulative frequency curves of LVOT-AR after TAVR for the five THVs. The shaded background shows the area above 17% of AR, indicating moderate or severe regurgitation. LVOT-AR: quantitative aortic regurgitation in the left ventricular outflow tract; TAVR: transcatheter aortic valve replacement; THV: transcatheter heart valve.

Among 181 aortograms of patients treated with the Venus A-valve, 113 (62.4%) were analyzable by quantitative assessment. The mean LVOT-AR was 8.9%±10.0% with 14.2% of the patients having a moderate or severe AR in the Venus A-valve group. For the other THVs, the lowest mean LVOT-AR was observed with the Evolut Pro (7.3 ± 6.5%, n = 95), followed by Evolut R (7.9 ± 7.4%, n = 295), Acurate Neo (9.6 ± 9.2%, n = 115), and CoreValve (13.7 ± 10.7%, n = 532) (Figures 2 and 3). No statistically significant difference in LVOT-AR was observed between Evolut Pro, Evolut R, Acurate Neo, and Venus A-valve, but a significantly higher LVOT-AR was observed with the CoreValve compared to the other four valves (Figure 2). The incidence of LVOT-AR >17% (moderate or severe regurgitation) with the Evolut Pro was significantly lower than with the Venus A-valve (5.3% vs. 14.2%, p = 0.034), the CoreValve (5.3% vs. 30.1%, p < 0.001), but was similar to that observed with the Evolut R (5.3% vs. 8.8%, p = 0.612) and Acurate Neo (5.3% vs. 11.3% p = 0.16). The Venus A-valve had a significantly lower rate of moderate or severe AR than the first generation CoreValve (14.2% vs. 30.1%, p = 0.001) (Figure 1).

**Figure 4:**
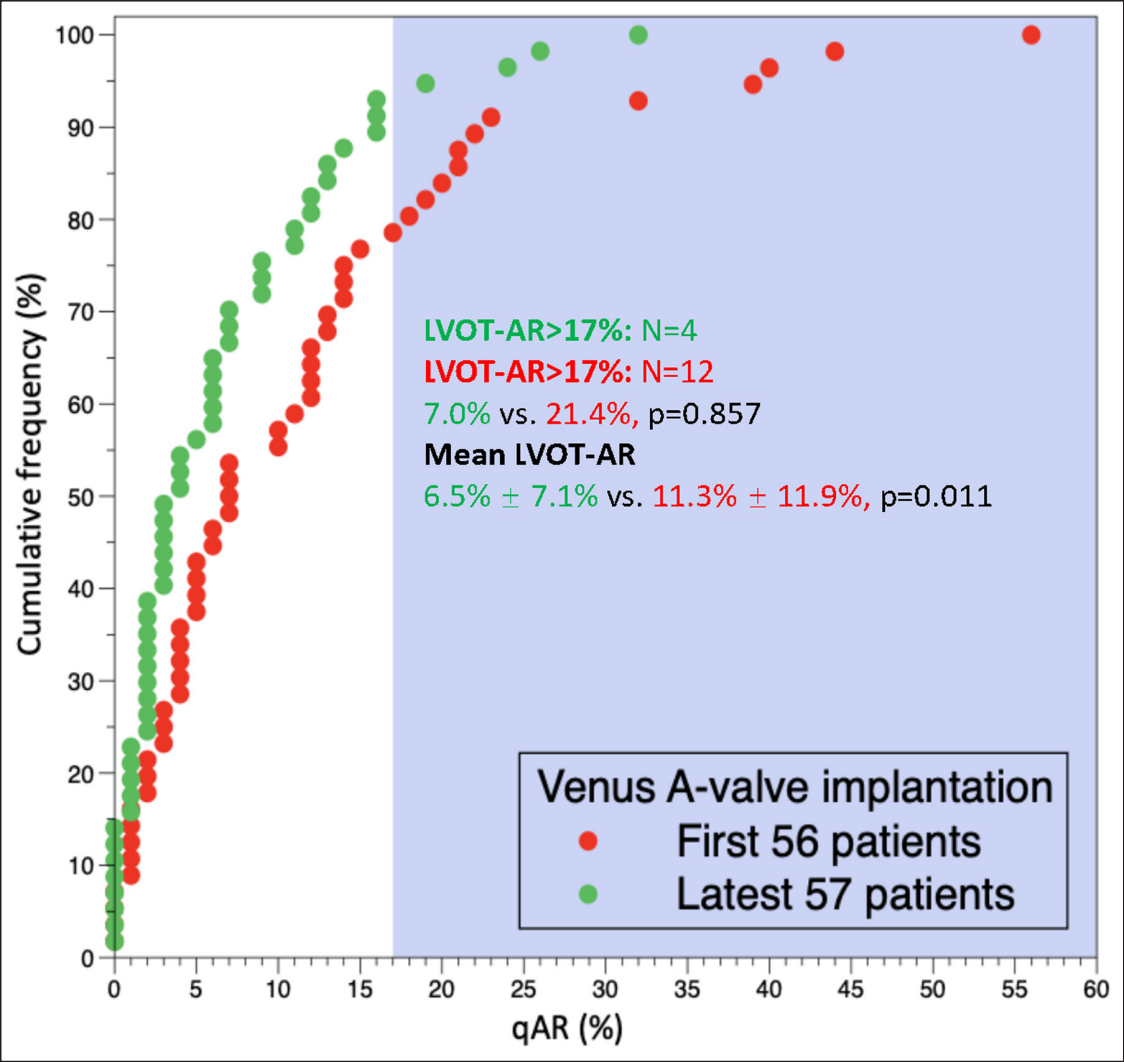
Cumulative frequency curves of LVOT-AR for the first 56 cases and the latest 57 cases with Venus A-valve. LVOT-AR: quantitative aortic regurgitation in the left ventricular outflow tract.

A landmark analysis of the first 56 Venus A-valve treated patients compared to the latest 57 cases (Figure 4) revealed a significantly lower mean LVOT-AR in the latest cases (11.3% ± 11.9% vs. 6.5% ± 7.1%, p = 0.011). As a binary parameter, the incidence of LVOT-AR >17%, in the latest 57 cases was numerically lower than the first 56 cases (7.0% vs. 21.4%, p = 0.857).

## Discussion

Paravalvular regurgitation is associated with mortality following TAVR, even in those with mild AR [10,16]. Accurate procedural assessment of AR is critical for the long-term success of TAVR. Aortic root angiography, typically using Seller’s visual grading [17], is the first minimalist assessment tool used in most laboratories for detection of paravalvular AR immediately post implantation and for guidance of timely corrective measures (e.g. post-dilation, valve-in-valve and, most recently, retrieval and reposition of the valve). However, the Seller’s classification of AR is a binary and non-continuous eyeball assessment, is subjective and thereby poorly reproducible. Although the evaluation of residual AR by transthoracic echocardiography (TTE) is still viewed as the gold standard, performing TTE in the cath-lab in a prone position is challenging and raises logistic and organizational issues [18].

Previous studies have demonstrated that TAVR performed exclusively with hemodynamic assessment and angiographic guidance with ‘back-up’ TTE is feasible and associated with reasonably good outcomes. It is similar to angiography and transesophageal echocardiography-guided procedures [19]. Based on the previous validation [8,13,14], quantitative aortographic assessment of AR has been shown to be an objective, accurate, and reproducible tool for assessment of AR following TAVR, and has the potential to facilitate timely decision-making to correct the AR with balloon post-dilatation, when additional corrective maneuvers are mandatory from a functional and prognostic perspective [15]. Conversely the online, objective and quantitative assessment also allows for the decision making to avert an unnecessary post-dilatation of the THVs that always entails the risk of a potential, manifest or silent, neurologic event.

In the current study, the Venus A-valve, in comparison with other commercially available SEVs analyzed in our database, showed a similar mean LVOT-AR. However, the occurrence of moderate or severe PVL was more frequently observed than with the Evolut Pro. This could be partially explained by the lack of external anti-leak skirt of the Venus A-valve on the valve frame. The external skirt contributes to minimizing AR by facilitating the plugging of micro-channels at the THV anchor site. To mitigate AR after TAVR, anti-leak skirt and pericardial wrap should be implemented in the development of the new generation SEV (Table 1). Another possible explanation for the difference is most likely due to the fact that the Venus A-valve is not repositionable (first generation) and therefore is frequently implanted low.

**Table 1:** Design features of the five THVs.

THVs	Self-expanding pericardial skirt	External pericardial wrap	Other features

**Evolut Pro** (23, 26, 29 mm)	Longer than CoreValve	yes	Porcine pericardium tissue valve. SE Nitinol frame. Recapturable, retrievable, repositionable. Ten percent smaller in height than CoreValve. 16 Fr.
**Evolut R** (23, 26, 29, 31 mm)	Longer than CoreValve	no	Porcine pericardium tissue valve. SE Nitinol frame. Recapturable, retrievable, repositionable. Ten percent smaller in height than CoreValve. 14/16 Fr.
**Venus-A** (23, 26, 29, 32 mm)	yes	no	Porcine native aortic leaflets. SE nitinol frame. Not retrievable, repositionable. Designed with increased radial force at the initial 20 mm of the stent inflow segment. Has three positioning marker. 19 Fr.
**Acurate Neo** (23, 25, 27 mm)	yes	no	Porcine native aortic leaflets. SE nitinol frame. Not retrievable, repositionable, fast pacing. Minimal protrusion into LV. Top-down deployment. The self-alignment and self-centering design. 18 Fr.
**CoreValve** (26, 29, 31 mm)	yes	no	Bovine pericardium tissue valve. SE Nitinol frame. Not recapturable, not retrievable, not repositionable. 18/20 Fr.

In addition, patients presenting for TAVR in China have a very high frequency of bicuspid valve morphology [20], which has been demonstrated to associate with a higher rate of PVL [21]. In the present cohort, 47 patients (41.6%) had bicuspid valve morphology. This anatomical feature of the native aortic valve in Chinese TAVR population may also contribute to the relative higher AR post TAVR procedures. Despite this possible anatomic drawback related specifically to Chinese patients, the TAVR operators of the Xijing hospital have improved results in the second half of the cohort, possibly after having overcome the learning curve with this THV. The present findings should be confirmed in prospective randomized comparisons of AR between different THVs.

## Limitations

First, only 62.4% of the Venus-A patients studied had aortograms that were suitable for video densitometric analysis. This is similar to previously reported rates from other retrospective series, while single-center studies abiding by a specific acquisition protocol, either with offline or online analysis, have analyzable data in 92.0% to 100.0% of cases [14,22,23]. Second, the data stem from a single-center without prior TAVR experience and had to overcome the discussed learning phase. Third, we present only data on the acute performance of the valve with either echocardiographic or angiographic (visual) assessments, and the long-term prognostic impact of LVOT-AR on clinical outcome is not available. Fourth, the comparison of the Venus A-valve with other SEVs is based on a retrospective, single-center experience and prospective, multi-center, randomized, head-to-head comparisons of these THV systems using a video densitometric imaging is warranted and indeed, is current ongoing (ClinicalTrials.gov NCT04275726).

## Conclusions

When compared to historic and current other commercially available self-expanding THVs, the Venus A-valve has comparable mean LVOT-AR as assessed by quantitative video densitometric measurement, but has a higher proportion of patients with moderate or severe AR than the Evolut Pro THV. After a learning curve, the incidence of moderate or severe AR compared favorably with the best in class currently available SEV. Additional developments of the Venus A system such as the addition of a sealing skirt and the ability to recapture and reposition the valve are likely to improve LVOT-AR performance.
